# Case Report: A successful case of toxic epidermal necrolysis treated with plasmapheresis therapy

**DOI:** 10.12688/f1000research.125050.1

**Published:** 2022-09-05

**Authors:** Wahyu Lestari, Vella Vella, Teuku Yasir, Teuku Zulfikar

**Affiliations:** 1 Department of Dermato-Venereology, Medical Faculty, Universitas Syiah Kuala, Banda Aceh, Aceh, 23111, Indonesia; 2 Department of Dermato-Venereology, Dr. Zainoel Abidin Hospital, Banda Aceh, Aceh, 24415, Indonesia; 3 Department of Anesthesiology, Medical Faculty, Universitas Syiah Kuala, Banda Aceh, Aceh, 23111, Indonesia; 4 Department of Anesthesiology, Dr. Zainoel Abidin Hospital, Banda Aceh, Aceh, 24415, Indonesia; 5 Department of Pulmonology, Medical Faculty, Universitas Syiah Kuala, Banda Aceh, Aceh, 23111, Indonesia; 6 Department of Pulmonology, Dr. Zainoel Abidin Hospital, Banda Aceh, Aceh, 24415, Indonesia

**Keywords:** toxic epidermal necrolysis, TEN, plasmapheresis, steroid, SCORTEN score

## Abstract

Toxic epidermal necrosis (TEN) is rare and can be life-threatening for patients. Appropriate management of TEN patients could give optimal results and prevent complications. One treatment modality for TEN is plasmapheresis, which is rarely available in most cases with severe TEN. Here we reported a successful treatment of severe TEN with plasmapheresis. A 40-year-old woman under tuberculosis therapy complained of shortness of breath that began four days prior to hospital admission and worsened ever since. The patient's skin was peeling with red spots and rashes all over the body for a week. During the examination, the patient was compos mentis, and the SCORTEN score was 2 with 12.1% risk of mortality rate. Dermatological examination of the face, trunk and extremities found extensive erosions, loose bullae filled with clear fluid, brown crusts, and generalized distribution with more than 30% epidermolysis. The patient was diagnosed with toxic epidermal necrolysis caused by antituberculosis therapy. We treated the patient by discontinuing the suspected drugs and administering the corticosteroids, but no improvement was observed. The patient underwent two cycle plasmaphereses with 5% albumin, resulting in 1.2 liter of plasma exchange. Re-epithelialization was observed after three days, and the patient was discharged on day 8. This case-report highlights the important role of plasmapheresis in treating the TEN patients. However, a study with larger sample sizes is warranted to validate the efficacy of plasmapheresis in TEN.

## Introduction

Toxic epidermal necrolysis (TEN), a potentially life-threatening condition, is an acute immune reaction characterized by necrosis and exfoliation of the epidermis covering more than 30% of the surface area of the body. Epidermolysis causes red spots to appear, which subsequently turn into blisters. ^1^^,^^2^ Because of the high mortality rate of TEN, a comprehensive management of the condition includes fast diagnosis, prompt identification of the causative medication, intensive care treatment, and prognosis evaluation using the Severity of Illness for TEN (SCORTEN). ^3^ Supportive therapy, such as isolation, maintaining fluid and electrolyte balance, nutrition, pain management, antimicrobials, and wound dressing are considered sufficient for TEN patients. However, there is uncertainty about the optimal systemic therapy to treat TEN. ^2^

Drug-induced TEN is usually caused by anticonvulsants, allopurinol, and antibiotics. ^4^ Phenytoin was the most common cause in the anticonvulsant group, while sulfamethoxazole-trimethoprim is the most common cause in the antibiotic group. ^4^ Antituberculosis agents have also been reported as the cause of TEN with isoniazid (INH) and streptomycin injection as the offenders. ^5^^–^^7^ However, TEN cases caused by antituberculosis agents were mostly reported in patients with immunosuppressant treatment, such as in HIV or SLE patients. ^7^^–^^9^ The timeline from presenting TEN symptoms to hospital admission usually varies from 4-15 days. ^7^^,^^8^ Here we report an immunocompetent patient with delayed TEN 30 days after antituberculosis therapy.

## Case presentation

A 40-year-old woman, Acehnese civil servant, presented to the Dr Zaionel Abidin Hospital in Banda Aceh Indonesia with shortness of breath. One week before the admission, the patient’s skin was peeling; red blotches and rashes emerged all over the body. The patient also reported that their eyes were itchy. The patient was coughing up phlegm for three days before admission. There was no fever and no documented allergy history.

The patient had the same complaints in 2011 and 2013 and was diagnosed with allergic contact dermatitis and Steven Johnson Syndrome (SJS). However, the patient was unable to recall the drug(s) that was associated with it. The patient had type 2 diabetes mellitus, and tuberculosis and was routinely treated with insulin Novorapid 12-12-12, Levemir 0-0-0-12 and tuberculosis drugs. The patient's condition worsened since the patient took tuberculosis drugs for one month.

**Figure 1.**
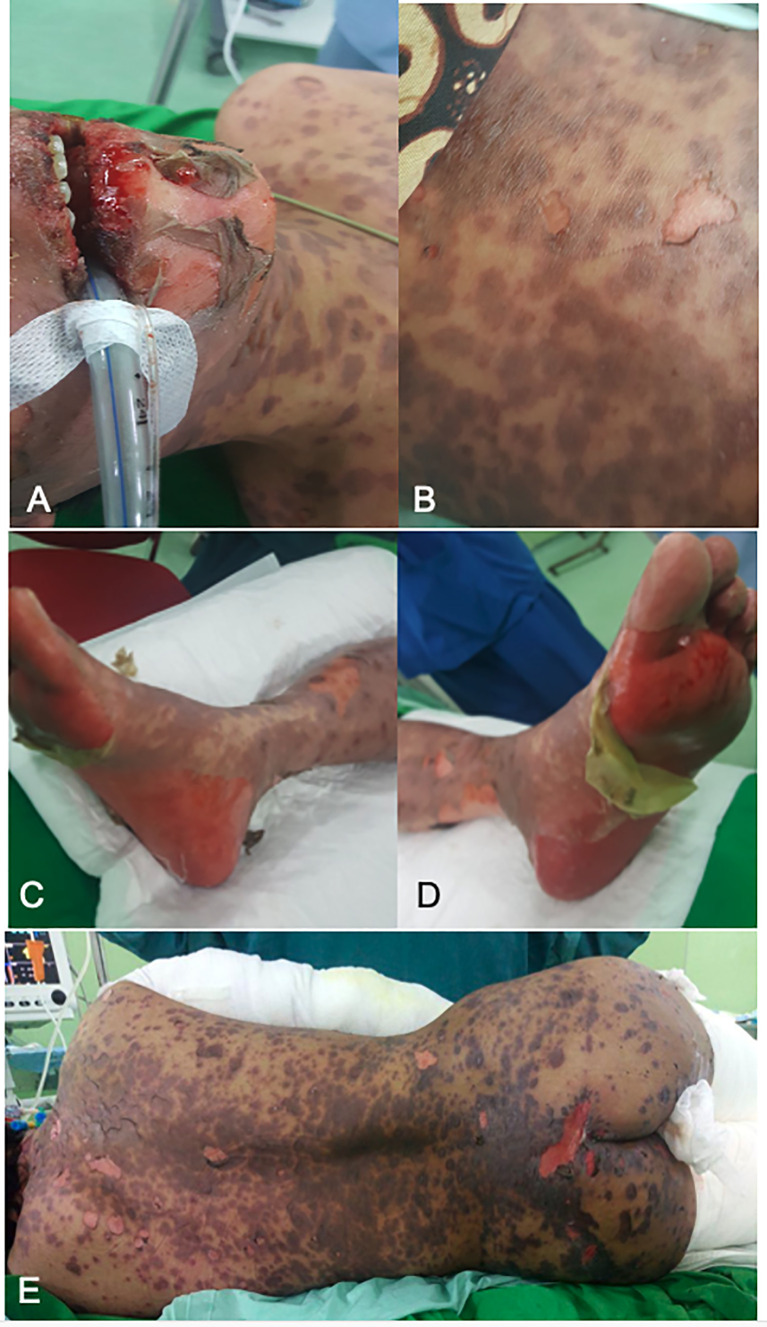
Skin abnormalities of patient before therapy in the facial and colli region (A), the trunk region (B), bilateral lower extremity et pedis region (C and D), posterior thoracic region (E) showed erythematous macules, bullae, erosions and brownish crusts with a positive Nikolsky sign.

On physical examination, eyelid edema, bulbar conjunctiva, and tarsal superior and inferior hyperemia were observed; the mouth was excoriated and fissured. The genital area was within normal limits. On pulmonary examination, there was a decreased tactile fremitus, decreased breath sound and ronchi over the left lung suggesting pleural effusion. Resonant percussion and auscultation were vesicular in the right lung field. On the dermatological status, macules, erythematous plaques, multiple brownish crusts, and nummular-size plaques were observed on regio of facialis, superior and inferior thoracic, and superior and inferior extremities with epidermolysis > 90% of body surface area, and Nikolsky sign (+) ( Figure 1). The SCORTEN score was used to calculate the prognostic score and yielded a score of 2 with a possible mortality rate of 12.1%.

The blood test showed hemoglobin 13.6 g/dL, platelets 302.000/mm ^3^, leukocytes increased by 16.000/mm ^3^. Kidney and liver function, as well as blood sugar levels within normal limits. Blood gas analysis showed pH 7.497 mmHg, pCO _2_ 29.00 mmHg, pO _2_ 61 mmHg, bicarbonate 22.7 mmHg, total CO _2_ 23.6 mmHg, BE 0.3 mmol/L, and oxygen saturation 93.5%.

Histopathological examination showed mild acanthosis, vacuolar changes, and scattered apoptotic keratinocytes in the epidermis, consistent with TEN. The dermis has a perivascular infiltrate consisting of lymphocytes, eosinophils, and neutrophils. There were no subcorneal spongiform and/or intraepidermal pustules.

Based on the physical examination and histopathology test result, the patient was diagnosed to have toxic epidermal necrolysis. The tuberculosis drug was discontinued because it was suspected the drug was the cause. Furthermore, the patient was taking oral prednisone and loratadine. After consultation to Department of Dermato-Venereology, the therapies were replaced by methylprednisolone injection 62.5 mg, oral mebhydrolin napadisylate 50 mg and combination of salicylic acid 3% and desoximethasone 0.25%, and vaseline album. Because there was no improvement observed, the patient was planned to undergo plasmapheresis with 5% albumin using the prismaflex machine. On the fourth day of plasmapheresis, the patient was given desoximethasone ointment as additional therapy. Methylprednisolone started to be tapped into 31.25 mg per day, fluconazole drip therapy200 mg/day and sun protection factor (SPF) 50 PA+ sunscreen was added on the fifth day.

**Figure 2.**
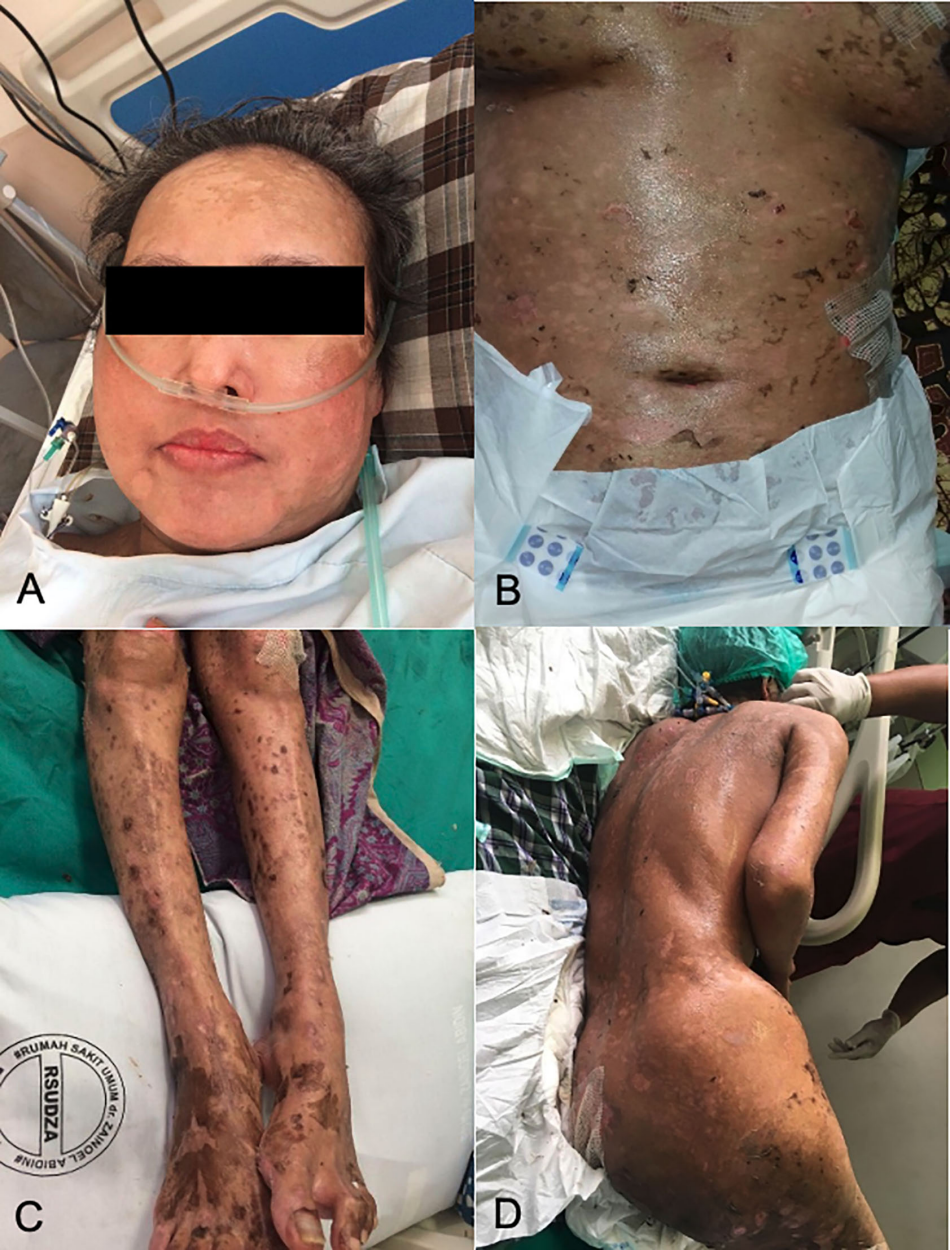
Recovery of the patient after the therapy. Purplish red macules were reduced two weeks after therapy in face region (A), abdominal (B), bilateral inferior extremity and pedis (C), and dorsum region (D).

After a week of therapy, the skin lesions improved, and the itchy skin complaints decreased. Purplish red macules with hypopigmented macules in the middle begin to diminish. Methylprednisolone injection therapy was replaced by oral treatment ( Figure 2). Re-epithelialization was observed after three days, and the patient was discharged on day eight.

## Discussion

TEN is an immediate reaction to a treatment marked by skin death and peeling in the epidermis. Based on the distribution of skin lesions, patients are classified to: (1) SJS (<10% body area surface), (2) SJS-TEN overlapping (10%-30% body area surface); and (3) TEN (>30% body area surface). Following the skin eruption, there was mucosal involvement in two areas: the eyes and the mouth. This occurs in nearly 80% of all TEN patients. ^1^^,^^10^ Shortness of breath indicates pulmonary problems. Early pulmonary problems occur in about 25% of patients and are primarily manifested by an increased respiratory rate and cough. Therefore, the presence of these three main symptoms leads to TEN diagnosis. ^10^^,^^11^ TEN symptoms usually develop after 1-3 weeks of the drug suspected of causing the symptoms is administered. ^12^ TEN symptoms usually appear between 6 days to 14 days after taking antituberculosis therapy in tuberculosis patients. ^13^^,^^14^ Das *et al.* reported a TEN case in an immunocompetent adult with tuberculosis, however, the patient was elderly (67-years-old). ^13^ The longest duration between drug administration and TEN symptoms presentation was five weeks. ^6^ Our case is the first case that reports TEN presentations in an immunocompetent adult patient without underlying comorbidities. The delayed reaction in our patient might be related to type IV hypersensitivity reaction, where T-cells mediate against specific drug/peptide antigens. ^15^ The manifestation of type IV hypersensitivity ranges from mild such as maculopapular exanthema, contact dermatitis or drug eruption to life-threatening SJS and TEN. ^16^ Genetic polymorphism of human leukocyte antigen (HLA) allele also plays an important role in inducing TEN, with HLA-C*04:01 significantly associated to antituberculosis drug-induced hypersensitivity. ^17^

There are no laboratory tests to support the diagnosis of TEN. Laboratory tests are performed to evaluate severity, prognosis, and daily management of such a life-threatening condition. In general, blood gas analysis indicates respiratory alkalosis and associated with bronchial involvement. Arterial blood gas analysis should be monitored because serum bicarbonate levels below 20 mEq/L indicate a poor prognosis. ^18^ Acute respiratory failure progressed rapidly after the onset of skin involvement and was associated with a poor prognosis. ^18^

Therapeutic management of TEN patients necessitates prompt identification and the withdrawal of the suspected substance as soon as possible, accompanied by supportive and customized therapy. There is no best-specialized therapy for TEN until now. ^19^ In this case, tuberculosis drugs were stopped temporarily.

Supportive care includes: maintaining fluid, electrolytes, optimal ambient temperature of 28-30°C, nutrition, aseptic skincare without debridement, and eye care. ^20^ Skin erosion in TEN patients causes hemodynamic problem. Nasogastric tube as a feeding route was used because the lesions on the oral mucosa make eating difficult or impossible. ^21^ The eyelids should be gently cleaned daily with a sterile isotonic sodium chloride solution. A study found that acute conjunctivitis preceded the skin eruption in 42 cases and co-occurred in 21 cases. ^21^ Topical ocular steroids early in the disease could result in a good visual prognosis. ^3^^,^^21^

The use of corticosteroids is still controversial. Corticosteroids can prevent the disease prolongation if given during the initial phase. ^3^ However, studies found that steroids did not stop disease progression and were linked with increased mortality and side effects, particularly sepsis. ^3^^,^^21^ Corticosteroids reduce epidermal apoptosis by numerous methods, including inhibition of different cytokines such as TNF-α, inhibition of INF-γ, which can promote apoptosis, and inhibition of Fas-mediated keratinocyte death. ^3^ In the absence of clinical trials, specific guidelines for the use of systemic corticosteroids vary and are not indicated as the primary treatment for epidermal necrolysis.

Plasmapheresis is exchanging the patient's plasma and replacing it with 5% albumin, FFP, colloids, or crystalloids. The use of plasmapheresis could release the circulating drugs as well as their metabolites or inflammatory mediators such as cytokines and it is one of the modalities of adjuvant therapy in TEN. The use of plasma exchange is a relatively safe intervention in more severe forms of TEN due to the presence of inflammatory cytokines, autoantibodies, expressed immune complexes or other toxic substances that cannot be removed by dialysis. From epidemiological studies of TEN throughout Japan from 2005 to 2007, plasmapheresis is now only used as a last resort in TEN patients who do not improve with standard therapy, including high-dose corticosteroids or adequate IVIG, or for patients with severe clinical complications such as encephalopathy hepatic and in patients with TEN involving more than 70% BSA. ^19^^,^^22^ Previous studies that reported the use of plasmapheresis in TEN patients are presented in Table 1. Of 119 patients, antibiotics triggered TEN in 19.3% cases, while NSAID medication caused TEN in 14.3% cases. Most patients were initially treated with corticosteroids (92/119, 77.3%), however, as no improvement was observed, plasmapheresis was included in the management of TEN (64.7%) in combination with intravenous immunoglobulin in 32.8% patients (39/119). The majority of the patients survived the disease (101/119, 84.9%) with fatality only involving 18 patients (15.1%). The use of plasmapheresis in TEN patients is justified due to its activity in removing the offending drug metabolites and other cytotoxic mediators from the circulation. ^23^ Treatment with plasmapheresis reduced the severity of TEN in patients, ^24^ however, the efficacy of plasmapheresis in toxic epidermal necrolysis should be further evaluated. ^19^^,^^22^

**Table 1.** The use of total plasma exchange in toxic epidermal necrolysis cases.

Study design	Total patients	Mucosa involved				Drug causing TEN								Outcome		TEN therapy				Ref	
		Oral	Genitalia	Eyes	Anal	Anticonvulsant	Antibiotic	Allopurinol	muscle relaxant	NSAID	CCB	ART	Immunomodulating agent	Lived	Died	Plasmapheresis	Steroid	IVIG	Antibiotics	
Case series	7	7	4	4	1	3	2	2	-	-	-	-	-	7	0	7	-	-	-	^27^
Retrospective	8	-	-	-	-	-	4	-	2	2	-	-	-	7	1	8	-	-	-	^28^
Case series	13	11	4	12	2	5	3	1	-	3	1	-	-	10	3	13	6	1	3	^29^
Case report	1	1	1	1	-	-	1	-	-	-	-	1	-	1	-	1	1	-	-	^30^
Case series	5	5	-	5	-	-	-	-	-	-	-	-	-	4	1	5	4	5	4	^31^
Case report	1	1	1	1	-	-	-	-	-	-	-	-	1	1	-	1	1	-	1	^32^
Case report	1	1	1	1	-	-	-	-	-	-	-	-	-	1	-	1	-	-	1	^33^
Case report	1	-	-	-	-	-	1	-	-	-	-	-	-	-	1	1	-	-	-	^34^
Case report	1	1	-	1	-	1	-	-	-	-	-	-	-	1	-	1	1	1	-	^35^
Case series	4	4	4	4	1	-	4	-	-	-	-	-	-	3	1	4	4	1	3	^36^
Case report	1	1	1	1	-	-	1	-	-	-	-	-	-	1	-	1	1	1	1	^37^
Case report	1	1	1	1	-	-	-	-	-	1	-	-	-	1	-	1	1	1	1	^38^
Case series	3	3	`1	3	-	-	-	2	-	-	-	-	-	3	-	1	3	3	-	^39^
Case report	1	1	1	1	-	-	-	-	-	-	-	-	-	1	-	1	1	-	-	^22^
Case report	1	1	1	1	1	-	1	-	-	-	-	-	1	-	1	1	1	1	1	^40^
Case report	1	1	-	1	-	-	1	-	-	-	-	-	-	-	1	1	1	1	1	^41^
Case report	1	1	1	1	1	-	-	-	-	1	-	-	-	1	-	1	1	-	1	^42^
Case report	1	1	1	1	1	1	-	-	-	-	-	-	-	1	-	1	1	1	1	^43^
Case report	1	1	1	1	-	-	-	-	-	1	-	-	-	1	-	1	-	1	1	^44^
Case report	1	1	1	1	1	-	-	-	-	1	-	-	-	1	-	1	1	1	1	^45^
Case series	3	3	3	3	-	-	-	-	-	-	-	-	3	3	-	3	3	3	3	^46^
Case report	1	1	1	-	-	-	-	-	-	1	-	-	-	1	-	1	1	-	-	^47^
Cohort study	59	36	27	32	-	4	5	-	-	7	-	-	-	51	8	19	58	16	-	^48^
case report	1	1	1	1	-	1	-	-	-	-	-	-	-	1	-	1	1	1	1	^49^
case report	1	1	1	1	11	-	-	-	-	-	-	-	1	-	1	1	1	1	1	^50^
	**119**	85	56	78	19	15	23	5	2	17	1	1	6	101	18	77	92	39	25	

ART: antiretroviral therapy; CCB: calcium channel blocker; IVIG: intravenous immune globulin; NSAID: nonsteroidal anti-inflammatory drug; PE: plasmapheresis.

There are some the limitations of our study. There was no challenge test on which tuberculosis therapy caused the TEN in our patient. Drug challenge in patients with a history of drug hypersensitivity remains the gold standard in diagnosing the drug tolerance. However, drug challenge is contraindicative in patients with severe cutaneous adverse reaction with severe organ involvement. ^25^ Therefore, combining *in vivo* (such as patch test and intradermal testing) and *ex vivo* methods (enzyme linked immunospot and lymphocyte transformation test) to investigate the suspected drug have been previously recommended. ^26^ Our study relies on a relatively limited number of databases to describe the effectiveness of plasmapheresis as a treatment for TEN. This case did not consider comparisons between plasmapheresis therapy and other alternative therapies. Long-term studies and follow-up with larger sample sizes are needed to validate the efficacy of plasmapheresis as a treatment for TEN.

## Conclusion

We report an immunocompetent patient with delayed TEN due to antituberculosis therapy with *SCORTEN score 2 and 12.1% risk of mortality rate. Two cycle plasmaphereses with 5% albumin resulting in rapid r*e-epithelialization and rapid recovery. This case-report suggests the important role of plasmapheresis in treating TEN patients.

## Consent

Written informed consent for publication of their clinical details and/or clinical images was obtained from the patient.

## Data availability

All data underlying the results are available as part of the article and no additional source data are required.
