# Supplementary material for: Case Report: A successful case of toxic epidermal necrolysis treated with plasmapheresis therapy
Source: F1000Res. 2022 Sep 5;11:995. [Version 1] doi: 10.12688/f1000research.125050.1 (PMC10148083; doi:10.12688/f1000research.125050.1)
Supplement: Peer review history

# Peer review: Reviewer response for version 1

**DOI:** 10.5256/f1000research.137313.r168692

**Published:** 2023-04-28

**Authors:** Nurdjannah Jane Niode

**Affiliations:** 1 Department of Dermatology and Venereology, Faculty of Medicine, Kandou General Hospital, Universitas Sam Ratulangi, Manado, Indonesia

In my opinion, this is an interesting case report which would be useful in practice for other practitioners, however I have a few suggestions regarding some points in the manuscript:

1. Is the background of the case’s history and progression described in sufficient detail?

 The introductory section was short and concise, however the authors may add the specificity/uniqueness related to the case. In this case the authors can bring up a little discussion about the treatment (plasmapheresis) in the introduction. Moreover, the objective of case reporting should be in line with the title. In the introduction, the objective doesn’t match the title.

2. Are enough details provided of any physical examination and diagnostic tests, treatment given and outcomes?

 In physical examination, it is better to explain the general condition of the patient and the vital signs (blood pressure heart rate, respiratory rate, temperature) should be stated, especially since heart rate is one of SCORTEN criteria.

 Regarding the treatment, I suggest it is better to mention whether the patient received supportive therapy (IV line) to maintain fluid and electrolyte balance, and if the authors were using trade name for drugs, please add ® sign behind the trade name, also the specific name of the anti-tuberculosis drugs should be written. The authors also didn’t mention any treatment regarding eye disorders in this case.

3. Is sufficient discussion included of the importance of the findings and their relevance to future understanding of disease processes, diagnosis or treatment?

 In this manuscript, the authors explain the relevant literature very well and in detail, however the authors need to add some explanations about the findings in this case and compare them with the relevant literature, not just discussing the literature. Furthermore, supportive care in discussion only exists in the literature but is not reflected in patient therapy either in case reports or discussions.

 4. In references, it is better if the authors cited from the literature with maximum of 10 years, unless there is no other reference can substitute it. Here is my suggestion of a few references that could be replaced: Reference No.2 (year 2000), No.27 (year 1997), No.28 (year 1999), 29, 30, 31, 32, 33, 34, 35. Is reference no. 19, 21 cited from books?

Are enough details provided of any physical examination and diagnostic tests, treatment given and outcomes?

Partly

Is the case presented with sufficient detail to be useful for other practitioners?

Yes

Is sufficient discussion included of the importance of the findings and their relevance to future understanding of disease processes, diagnosis or treatment?

Partly

Is the background of the case’s history and progression described in sufficient detail?

Partly

Reviewer Expertise:

Dermatology and venereology, Medical entomology

I confirm that I have read this submission and believe that I have an appropriate level of expertise to confirm that it is of an acceptable scientific standard.

# Peer review: Reviewer response for version 1

**DOI:** 10.5256/f1000research.137313.r156678

**Published:** 2022-12-05

**Authors:** Talha Bin Emran

**Affiliations:** 1 Department of Pharmacy, BGC Trust University Bangladesh, Chittagong, Bangladesh

I read the case report with great interest. The case is unique and has adequate description and analysis. Here are detailed evaluations:

1. The title is clear, with a focus on the treatment of the case.
2. The abstract consists of an introduction of the case, the main clinical findings, and the intervention, followed by a balanced conclusion.
3. The introduction is brief but still adequate and explains why the case is important.
4. The patient information is not provided, to follow the ethical issue. The figures are clear without personal information.
5. The description of the case is adequate, including the follow-up.
6. Some examinations were conducted to support the diagnosis. But the histopathological examination would be beneficial to readers if provided in the manuscript.
7. The discussion is balanced. The authors also discussed the molecular aspect.
8. One of the strengths of the study: the authors provided a literature review of the outcome of total plasma exchange in toxic epidermal necrolysis cases.
9. Minor typo: Some conclusion parts should be non-italic: “CORTEN score 2 and 12.1% risk of mortality rate. Two cycle plasmaphereses with 5% albumin resulting in rapid recovery. ...”

Are enough details provided of any physical examination and diagnostic tests, treatment given and outcomes?

Yes

Is the case presented with sufficient detail to be useful for other practitioners?

Yes

Is sufficient discussion included of the importance of the findings and their relevance to future understanding of disease processes, diagnosis or treatment?

Yes

Is the background of the case’s history and progression described in sufficient detail?

Yes

Reviewer Expertise:

Clinical Biochemistry

I confirm that I have read this submission and believe that I have an appropriate level of expertise to confirm that it is of an acceptable scientific standard.
